# A Game Theoretical Model for Location of Terror Response Facilities under Capacitated Resources

**DOI:** 10.1155/2013/742845

**Published:** 2013-12-28

**Authors:** Lingpeng Meng, Qi Kang, Chuanfeng Han, Weisheng Xu, Qidi Wu

**Affiliations:** ^1^Department of Control Science and Engineering, Tongji University, Shanghai 201804, China; ^2^Institute of Urban Construction and Emergency Management, Tongji University, Shanghai 200092, China

## Abstract

This paper is concerned with the effect of capacity constraints on the locations of terror response facilities. We assume that the state has limited resources, and multiple facilities may be involved in the response until the demand is satisfied consequently. We formulate a leader-follower game model between the state and the terrorist and prove the existence and uniqueness of the *Nash* equilibrium. An integer linear programming is proposed to obtain the equilibrium results when the facility number is fixed. The problem is demonstrated by a case study of the 19 districts of Shanghai, China.

## 1. Introduction

Different from regular natural calamity such as earthquakes, terrorist attacks have low frequency but tremendous magnitude [1]. As a result, the attacks can lead to a big demand for medical supplies, which overwhelm the local emergency responders, and regional/national assistance will be required. Moreover, it contains human intelligence and intent in terrorist attacks, just as the 9/11 Commission Report states, “The terrorists analyze defenses. They plan accordingly.” The interaction between the government and the terrorist increases the complication in the issue of counterterrorism.

Faced with terrorist attacks, implementing an efficient disbursement of the requisite emergency resources is critical in reducing morbidity and mortality; therefore, the need to be prepared to respond to the terrorist attacks is highlighted. Consequently, there is increasing interest in strategic facility location for protecting assets of value (including human lives) against terrorist attacks. Facilities that contain resources required to respond to terror attacks are referred to as terror response facility [2]. This paper will address the location problem of terror response facility under capacitated resources.

Location problem of emergency service facilities such as ambulance, police, and fire-fighting assistance has been previously investigated, and many models and their extensions have been developed to formulate and solve the problem, including covering models, *p*-median models, *p*-center models, and their extensions, for example, [3–6]. One could see [7, 8] for recent reviews. Such emergency facility location models (covering models, *p*-median models, *p*-center models, and their extensions) typically have not considered the attacker's analysis and response to the defender's strategy and tend to focus more on emergency response than on prevention. Many literatures address the strategic nature of facility location problems by modeling either stochastic or dynamic integer programming formulations, but as empirical data are likely to be sparse, classical statistical methods have been of relatively little use. The distinction between probabilistic and strategic risks has emerged recently, see; [9–11] and so forth. Snyder [12] provides a complete review on facility location problems with uncertainties. Unfortunately, most concentrate on common situations and few focus on conditions with a tremendous demand and low frequency which may occur in terror emergencies.

In addition, one critical assumption in most literatures is that the assigned facility can meet all the demands, which is not always the case for terrorist attacks. In fact, the state have not enough resources to cover all potential targets; thus situations with insufficient supplies will occur facing large uncertain demands. How to allocate the limited resources among the terror response facilities is another challenge besides the facility location.

Game theory is an appropriate tool for investigating counterterrorism as it captures the strategic interactions between terrorists and targeted state whose choices are interdependent. With respect to applications of game theory and related methods to security in general, there is a large number of work already, that is, to inform policy-level decisions such as public versus private funding of defensive investments [13] or the relative merits of deterrence and other protective measures, for instance, [14–17]; however, few has gone deep into operational level.

Both empirical and theoretical analysis showed that raising a potential target's defense level will increase the probability for another target to be attacked, and limited resources should be allocated to maximize the overall utility [18, 19]. The framework of game theory can effectively deal with resource allocation problems [20]. The efficiency and equity of capacitated resource allocation problems have been studied [21]. It is verified that making decision in global manner is superior to that in decentralized manner when allocating resources between two targets [22]. Powell [23] proposed a two-stage game between the terrorist and the state, to allocate capacitated resources between two targets, and extended the model to the case of *N* targets. Based on this analysis framework, some more points were studied, including the effect of the state's information [24], the interaction of more variables [25], the attacking behavior of the terrorist [10], and preassessment for utility of both sides [26]. Some correct but counterintuitive conclusions were deduced. First, as the arms race, the attack of the terrorist is positively correlated with the defense level of the state; second, the state has first-mover advantage in the sense that the state can force the terrorist to attack a target with smaller vulnerability; third, it will benefit the state to make the resource allocation policies open; finally, some targets with less loss for the state will be left unprotected under capacitated resources.

In a game-theoretical model, the introduction of capacity constraint will usually change the equilibrium results, even when the initial model is simple [27]. The pure equilibrium may be replaced by mixed equilibrium, or there will no equilibrium, as a result the complication of solution will increase [28]. At the same time, the change of the rationing rule, that is, the allocation of residual demand when one facility could not satisfy the whole demand, will probably change the equilibrium results [29]. We will solve the Nash equilibrium of the problem using an integer program, that could simplify the solution of the game.

To our knowledge, the only rigorous model to date to deal with the terror response facility problem is exploratory analyses by Berman and Gavious [2] and Berman et al. [30]. The state decides where to locate the terror response facilities, and the terrorist decides which node to strike with complete or incomplete information of the distribution of the facilities. Both papers assume that there is no capacity constraints on the facilities; that is, the facility could provide enough resource to the nearest node attacked. We will extend this assumption to the case that the state has capacitated resources, and he has to decide how to allocate the resources to each facility besides the location of facilities taking into consideration the behavior of the terrorist.

This paper assumes that the state has capacitated resources and could not satisfy all the demand simultaneously. When the facility nearest to the attacked city could not meet the demand, the second nearest facility will be involved in the rescue, and so on, until the required resources are satisfied. The state could spread the limited resource among more facilities to improve the overall coverage or adopt a centralized manner, among fewer facilities to protect key areas. A balance between resource quantity and allocation manner must be considered carefully.

The next section will formulate the problem. In Section 3, the Nash equilibrium of the game is discussed, and a simple example is given.  Section 4 proposes a linear integer programming to solve the equilibrium when the facility number is fixed. A case study is given in Section 5. Finally, we give conclusions in Section 6.

## 2. Problem Formulation

Consider an undirected connected network *G*(*V*, *E*), where *V* = (*v*_1_, *v*_2_,…, *v*_*n*_) is the set of nodes, and *E* = (*e*_1_, *e*_2_,…, *e*_*m*_) is the set of edges. The nodes are demand cities, and each city *v*_*i*_ is associated with a weight *ω*_*v*_*i*__ to represent the amount of resources needed every unit time. The shortest distance between node *v*_*i*_ and *v*_*j*_ is *d*[*v*_*i*_, *v*_*j*_], representing the delay of shipment of resources from *v*_*j*_ to *v*_*i*_. Let *R* = {*r*_1_, *r*_2_,…, *r*_*m*_} be the potential locations of the terror response facilities, *m* ≤ *n*. Without loss of generality, let *R* ⊂ *V*.

It is assumed that the resources needed of city *v*_*i*_ is *ω*_*v*_*i*__. Let the total resources of the state be a constant *C*, max⁡{*ω*_*v*_*i*__} ≤ *C* < ∑_*i*=1_^*n*^ *ω*_*v*_*i*__; that is to say, the state could satisfy the demand of every single city but could not eliminate the loss of terrorist attacks completely by establishing terror response facilities at each city. The resources associated with each facility *r*_*j*_, *j* = 1,2, 3,…, *m* are *c*_*i*_. For simplicity, we consider the symmetric condition; that is, *c*_1_ = *c*_2_ = ⋯ = *c*_*m*_ = *c*. Obviously, *C* = *mc*.

Once the city *v*_*i*_ is attacked, the nearest terror response facility will begin to provide the resources. If the nearest facility could not satisfy the demand, that is, *c* < *ω*_*v*_*i*__, the second nearest facility will be involved in the rescue, and so on. Let

(1) $$\begin{array}{c} D^{\left(v_{i}\right)}=\left\{d\left[v_{i},r_{1}\right],\ldots ,d\left[v_{i},r_{m}\right]\right\}, \end{array}$$

in which when *i* < *j*, *d*[*v*_*i*_, *r*_*i*_] < *d*[*v*_*i*_, *r*_*j*_]. The facilities will be involved in the rescue sequentially form *r*_1_ to *r*_*m*_. Let *p* = ⌈*ω*_*v*_*i*__/*c*⌉ be the smallest integer not less than *ω*_*v*_*i*__/*c* then *p* facilities will be needed.

As Berman and Gavious [2], the players of the game are the state and the terrorist. We assume that the loss of the state *f*_*G*_ is a linear function of the time delay of the resources,

(2) fG=(d[vi,r1]+⋯+d[vi,rp−1])·c+d[vi,rp]·(ωvi−(p−1)c).

The utility of the terrorist is the loss of the state,

(3) $$\begin{array}{c} U_{T}=f_{G}. \end{array}$$

We consider a zero-sum game; then the utility of the state is *U*_*G*_ = −*U*_*T*_.

The interaction is modeled as a sequential game with complete information. The state acts first. He must decide the resource allocated to each facility and the facility location such that the city attacked is the one that minimizes his disutility. The terrorist plays second and chooses a city to attack to maximize his utility observing the arrangement of the state.

## 3. Nash Equilibrium Solutions

The strategy of the state is *S*_*G*_ = (*c*, *R*), and the strategy of the terrorist is *S*_*T*_ = (*v*_*i*_). A Nash equilibrium mean that, for  all  (*S*_*G*_ and *S*_*T*_), we have

(4) $$\begin{array}{c} U_{G}\left(S_{G}^{*},S_{T}^{*}\right)\geq U_{G}\left(S_{G},S_{T}^{*}\right),\\ U_{T}\left(S_{G}^{*},S_{T}^{*}\right)\geq U_{T}\left(S_{G}^{*},S_{T}\right). \end{array}$$

Obviously, the equilibrium is also the subgame perfect Nash equilibrium of the game.

### 3.1. Facility Number is Fixed

When the facility number *m* is a constant, the resources *c* of every facility are fixed. Then, the strategy of the state is *S*_*G*_ = *R* = (*r*_1_,…, *r*_*m*_), and the strategy of the terrorist is *S*_*T*_ = *v*(*R*).

Given the state's strategy *R*, the best response of the terrorist is

(5) v∗(R)=arg⁡max⁡vi(d[vi,r1]+⋯+d[vi,rp−1])·c+d[vi,rp]·(ωvi−(p−1)c).

Forecasting the reaction of the terrorist, the state will adopt the strategy

(6) R∗=arg⁡min⁡R⊂V (d[v∗,r1]+⋯+d[v∗,rp−1])·c+d[v∗,rp]·(ωv∗−(p−1)c).

It is assumed that the storage and distribution manner of the resources is similar to the Strategic National Stockpile (SNS) of Centers for Disease Control and Prevention (CDC), USA. That is to say, the resource *c* at every facility will be shipped to the city attacked as a whole when the facility is involved in the rescue. In fact, this manner would increase the redundancy of resource supply and is more suitable for terrorist attacks.

**Lemma 1**

When the facility number is fixed, the state equilibrium solution is the solution of a minimax problem in the nodal set, and the terrorist equilibrium solution could be obtained by (5).

**Proof**

For any strategy of the state *R*, the best response of the terrorist is (5), and the best strategy of the state is decided by (6). From the definition of *p*, *ω*_*v*_*i*__/*c* ≤ *p* < *ω*_*v*_*i*__/*c* + 1. Let *f*_*G*_′ = (*d*[*v*_*i*_, *r*_1_] + ⋯+*d*[*v*_*i*_, *r*_*p*_]) · *c*; then *f*_*G*_′ = *f*_*G*_ + *d*[*v*_*i*_, *r*_*p*_](*pc* − *ω*_*v*_*i*__). Apparently, 0 ≤ *pc* − *ω*_*v*_*i*__ < *c*; (6) could be rewritten accordingly as

(7) $$\begin{array}{c} R^{*}=\arg\underset{R\subset V}{\min}\left(\underset{v_{i}}{\max}\;\left(\left(d\left[v^{*},r_{1}\right]+\cdots +d\left[v^{*},r_{p}\right]\right)\cdot c\right)\right). \end{array}$$

Compared with (6), (7) could simplify the solution of the equilibrium to some extent but will not change the behavioral characteristics of the state and the terrorist. The equilibrium of the terrorist could be obtained by substituting (7) into (5).

### 3.2. Facility Number is Variable

When the facility number is variable, if there are no capacity constraints, the optimal facility number is obviously *n*. With the capacity constraints, one facility could not satisfy the demand, and multiple facilities will be involved. As *m* increases, the resources at each facility will decrease, and more facilities may be involved. Smaller *m* corresponds to the centralized mode, in which the state will distribute the limited resources to protect the key areas, while larger *m* corresponds to the dispersed mode, in which the state will distribute the resources to get larger coverage. Different *m* will induce different disutility, and the state must strike a balance between the two mode. The relationship between the utility of the state and the parameters *c* and *m* is demonstrated by the following lemmas.

**Lemma 2**

When *c* is a constant, the equilibrium solution of the state ${\tilde{U}}_{G}(R^{*},v^{*}(R))$ is a monotonically increasing function of *m*.

**Proof**

From (2) and (3), ${\tilde{U}}_{G}(R^{*},v^{*}(R))=-((d[v^{*},r_{1}]+\cdots +d[v^{*},r_{p-1}])\cdot c+d[v^{*},r_{p}]\cdot (\omega_{v^{*}}-(p-1)c))$. When *c* is fixed, ${\tilde{U}}_{G}$ is a monotonically increasing function of *d*[v*, *r*_*i*_]. As *m* increasing, there must exist a *d*′[v*, *r*_*i*_] ≤ *d*[v*, *r*_*i*_], such that for the new utility function ${\tilde{U}}_{G}^{\prime }$, ${\tilde{U}}_{G}^{\prime }\geq {\tilde{U}}_{G}$. The proof is completed.

**Lemma 3**

When *m* is a constant, the equilibrium solution of the state ${\tilde{U}}_{G}(R^{*},v^{*}(R))$ is a monotonically increasing function of *c*.

**Proof**

From *p* = ⌈*ω*_v*_/*c*⌉, when *c* increases to *c*′, there will be *p*′ = (*ω*_v*_/*c*′) ≤ *p*. Two conditions will be discussed.

When *p*′ = *p*,

(8) U~G−U~G′ =−(d[v∗,r1]+⋯+d[v∗,rp−1])·c−d[v∗,rp]  ·(ωv∗−(p−1)c)+(d[v∗,r1]+⋯+d[v∗,rp−1])  ·c′+d[v∗,rp]·(ωv∗−(p−1)c′) =(d[v∗,r1]+⋯+d[v∗,rp−1])  ·(c′−c)+d[v∗,rp]·(p−1)(c−c′) =(d[v∗,r1]+⋯+d[v∗,rp−1]−(p−1)d[v∗,rp])  ·(c′−c)<0.

Accordingly, ${\tilde{U}}_{G}<{\tilde{U}}_{G}^{\prime }$.

When *p*′ < *p*, we just need to prove the correctness of the conclusion when *p*′ = *p* − 1. Consider

(9) U~G−U~G′ =−(d[v∗,r1]+⋯+d[v∗,rp−1])  ·c−d[v∗,rp]·(ωv∗−(p−1)c)  +(d[v∗,r1]+⋯+d[v∗,rp−2])  ·c′+d[v∗,rp−1]·(ωv∗−(p−2)c′) =(d[v∗,r1]+⋯+d[v∗,rp−2])  ·(c′−c)+d[v∗,rp−1]·(ωv∗−(p−1)c′−c)  −d[v∗,rp]·(ωv∗−(p−1)c) <(d[v∗,r1]+⋯+d[v∗,rp−2])  ·(c′−c)+d[v∗,rp−1]·(ωv∗−(p−1)c′−c)  −d[v∗,rp−1]·(ωv∗−(p−1)c) =(d[v∗,r1]+⋯+d[v∗,rp−2])·(c′−c)+d[v∗,rp−1]  ·((p−1)(c−c′)−c) <(d[v∗,r1]+⋯+d[v∗,rp−2])  ·(c′−c)+d[v∗,rp−1]·(p−1)(c−c′) =(d[v∗,r1]+⋯+d[v∗,rp−2]−(p−1)d[v∗,rp−1])  ·(c′−c)<0.

Accordingly, ${\tilde{U}}_{G}<{\tilde{U}}_{G}^{\prime }$. The proof is completed.

Evidently, the facility number *m* and the resources *c* at each facility will affect the equilibrium solutions simultaneously; hence, it is not so straightforward to decide the optimal facility number and the location. However, when the total resources of the state *C* are constant, there is an inversely proportional relationship between *c* and *m*, so we could compare the disutility of the state under different facility numbers, and the optimal number is the one that induces the smallest disutility. We could get the following theorem.

**Theorem 4**

There exists a unique c*, such that {*S*_G_* = {c*, R*}, *S*_T_* = {v*}} is the Nash equilibrium solution of the game, and the optimal *m* could be obtained by

(10) $$\begin{array}{c} m^{*}=\arg\underset{m=1,\ldots ,n}{\max}\;U_{G}\left(R^{*},v^{*}\left(R\right)\right). \end{array}$$

### 3.3. An Example

**Figure 1:**
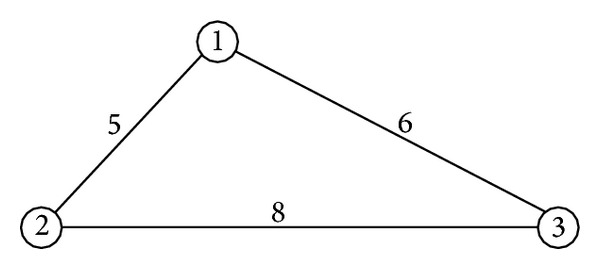
The network in the example.

We will use a simple example to demonstrate the solution of the equilibrium. Consider the network in Figure 1, in which the numbers next to the edges are the distance between node pairs; that is, *d*[1,2] = 5, *d*[2,3] = 8, *d*[1,3] = 6. Let *ω*_1_ = 16, *ω*_2_ = 10, *ω*_3_ = 21 and *C* = 24.

When *m* = 1, *c* = 24, the strategy of the state is *R* = {1,2, 3}. From (5), the best responses of the terrorist are

(11) $$\begin{array}{c} v^{*}\left(1\right)=\arg\max\left\{16\times 0,10\times 5,21\times 6\right\}=3,\\ v^{*}\left(2\right)=\arg\max\left\{16\times 5,10\times 0,21\times 8\right\}=3,\\ v^{*}\left(3\right)=\arg\max\left\{16\times 6,10\times 8,21\times 0\right\}=1. \end{array}$$

From (6), the state equilibrium is

(12) R1∗=arg⁡min⁡⁡{UG(1,3),UG(2,3),UG(3,1)}=arg⁡min⁡⁡{126,168,96}=3.

The equilibrium solution of the game is {3,1}, and the utility of the state is *U*_*G*1_* = −96.

When *m* = 2, *c* = 12, the strategy of the state is *R* = {(1,2), (2,3), (1,3)}, and the best responses of the terrorist are

(13) v∗(1,2) =arg⁡max⁡⁡{12×0+4×5,10×0,12×6+9×8}=3,v∗(1,3) =arg⁡max⁡⁡{12×0+4×6,10×5,12×0+9×6}=3,v∗(2,3) =arg⁡max⁡⁡{12×5+4×6,10×0,12×0+9×8}=1.

The state equilibrium is

(14) R2∗=arg⁡min⁡{UG((1,2),3),UG((1,3),3),UG((2,3),1)}=arg⁡min⁡{144,54,84}=(1,3).

The equilibrium solution of the game is {(1,3), 3}, and the utility of the state is *U*_*G*2_* = −54.

When *m* = 3, *c* = 8, the strategy of the state is *R* = {(1,2, 3)}, and the state equilibrium is *R*_3_* = (1,2, 3). The best response of the terrorist is

(15) v∗(1,2,3)=arg⁡max⁡⁡{8×0+8×5,8×0+2            × 5,8×0+8×6+5×8}=3.

The equilibrium solution of the game is {(1,2, 3), 3}, and the utility of the state is *U*_*G*3_* = −88.

From (10), the optimal *m* is m* = arg⁡max⁡_*m*=1,2,3_{*U*_*G*1_*, *U*_*G*2_*, *U*_*G*3_*} = 2. The Nash equilibrium of the game is ((12, (1,3)), 3). The state will establish terror response facilities at node 1 and node 3, and the terrorist will attack node 3. The resource at each facility is 12, and the state will get a disutility of 54.

## 4. The Integer Programming

To solve the equilibrium efficiently, we formulate the game when the facility number is fixed as an integer program.

Define the decision variables as follows:

(16) yij={1,if  the  city  vi is  assigned  to  facility rj0,otherwise                ∀i,j=1,2,…,n,xj={1,if  a  facility  is  established  at  city  vj0,otherwise                ∀j=1,2,…,n,xj={1,if  the  terrorist  attacks  city  vi0,otherwise              ∀j=1,2,…,n.

Let *Q* be the terrorist utility in the equilibrium; then

(17) ∑i=1n[(d[vi,r1]yi1+⋯+d[vi,rp−1]y1,(p−1))  ·c+d[vi,rp]yip·(ωvi−(p−1)c)]zi≤Q.

At the same time, the state will minimize his disutility; that is,

(18) ∑i=1n[(d[vi,r1]yi1+⋯+d[vi,rp−1]y1,(p−1))  ·c+d[vi,rp]yip·(ωvi−(p−1)c)]zi≥Q.

Consequently, when the facility number is fixed, we could simplify (17) and (18) using (7).

The game could be formulated as follows:

(19) $$\begin{array}{c} \min Q \end{array}$$

s.t.

(20) $$\begin{array}{c} \overset{n}{\underset{j=1}{\sum }}y_{ij}=p_{i}\;\forall i\in I, \end{array}$$

(21) $$\begin{array}{c} m\frac{\omega_{v_{i}}}{C}\leq p_{i}<m\frac{\omega_{v_{i}}}{C}+1, \end{array}$$

(22) $$\begin{array}{c} \overset{n}{\underset{j=1}{\sum }}x_{j}=m, \end{array}$$

(23) $$\begin{array}{c} \overset{n}{\underset{i=1}{\sum }}z_{i}=1, \end{array}$$

(24) $$\begin{array}{c} y_{ij}\leq x_{j},\;\forall i,j=1,\ldots ,n, \end{array}$$

(25) ∑k=1nyijd[vi,vk]+∑l=1p(M−d[vi,rl])xl≤pM,              ∀i,j=1,2,…,n,

(26) $$\begin{array}{c} \overset{n}{\underset{i=1}{\sum }}\overset{n}{\underset{j=1}{\sum }}\left(d\left[v_{i},v_{j}\right]y_{ij}\frac{C}{m}\right)z_{i}=Q, \end{array}$$

(27) $$\begin{array}{c} \overset{n}{\underset{j=1}{\sum }}d\left[v_{i},v_{j}\right]y_{ij}\frac{C}{m}\leq Q,\;\forall i\in I, \end{array}$$

(28) $$\begin{array}{c} x_{j}\in \left\{0,1\right\},\;z_{i}\in \left\{0,1\right\},\;y_{ij}\in \left\{0,1\right\},\;p_{i}\in N^{+}, \end{array}$$

where *M* is a sufficiently large number, *M* ≥ *p* · max⁡_*v*_*i*_,*v*_*j*_∈*V*_ *d*[*v*_*i*_, *v*_*j*_].

Equations (20) and (21) are used to ensure that each city is assigned to enough facilities while not wasting resources. Equation (22) guarantees that the facility number is *m*. In (23), we make sure that the terrorist will attack only one city. In (24), we make sure that each city is assigned to a node with a facility built at it. In (25), we verify that when the nearest facility could not meet the demand, the second nearest facility will be involved, and so on. In (26) and (27), we take the state and the terrorist objectives into account. Binary requirements are specified in (28).

Obviously, the program is a nonlinear programming because the formulation 10 has multiplications of variables. We could define a new variable *u*_*ij*_ to linearize it. Let

(29) $$\begin{array}{c} u_{ij}=y_{ij}z_{i}, \end{array}$$

where

(30) $$\begin{array}{c} y_{ij}+z_{i}\leq 1+u_{ij}\;i,j=1,\ldots ,n,\\ y_{ij}+z_{i}\geq 2u_{ij}\;i,j=1,\ldots ,n. \end{array}$$

Then, (26) could be rewritten as

(31) $$\begin{array}{c} \overset{n}{\underset{i=1}{\sum }}\overset{n}{\underset{j=1}{\sum }}d\left[v_{i},v_{j}\right]u_{ij}\frac{C}{m}=Q. \end{array}$$

The linearized formulation of the game could be obtained by substituting (29)–(31) in (26).

## 5. Case Study

We solve the problem of terror response facility location under capacity constraints with a case study of districts in Shanghai, China. We assume that the resources needed are proportional to the population sizes of the districts. The 19 districts and their population sizes (10^4^) are given in Table 2. The shortest distance matrix between the districts is given in Table 3. Without loss of generality, we considered three scenarios for resource capacity of the state, that is, insufficient, moderate, and abundant, which correspond to *C* = 200,750,1300, respectively. The equilibrium solutions were obtained by the linear programming in Section 4 using the optimization software Cplex 12.2. The software was run on an Intel Core2 Duo 2.0 GHz processor with 1.0 GB RAM under Windows XP.

As stated above, different facility numbers could affect the state disutility, because the state could disperse the resources into more facilities to improve the coverage or could concentrate the resources to fewer facilities to protect the key areas. The state disutilities for different facility numbers are given in Table 1. From (10), the optimal facility number is 7, 10, and 13, respectively, when the total resources are 200, 750, and 1300, and the disutility is 3330, 1680, and 600.6. It shows that as the total resources increasing, the state could improve the coverage by establishing more facilities; at the same time, the resources at each facility will increase, that reduces the state disutility effectively.

**Figure 2:**
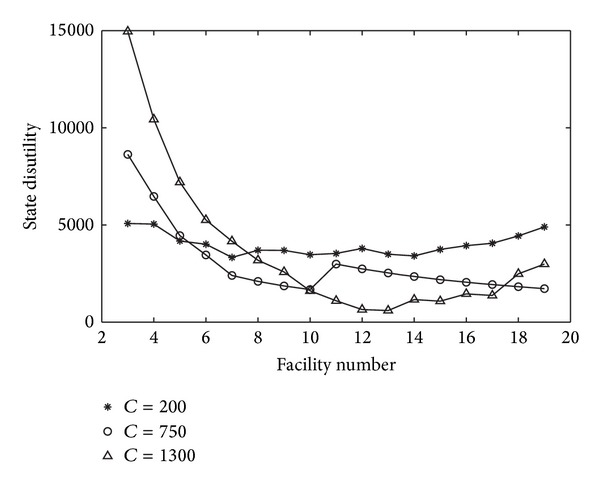
The relationship between facility number and the state disutility.

In Figure 2, we depict the disutility of the state for different facility number under the three scenarios. Obviously, when the total resources are fixed, it is not a simple linear relationship between the state disutility and the facility number. For example, in the abundant scenario, when *m* = 9, there are 144.4 resources at each facility, the state disutility is almost the same when *m* = 18, in which there are 72.2 resources at each facility. However, when *m* = 13, there are 100 resources at each facility, and the state disutility is much less than the two cases above. It implies that, distributing the resources in limited facilities could protect key areas but will reduce the coverage, while distributing the resources in more facilities will increase the shipment cost although the coverage is enhanced. A balance should be achieved.

**Figure 3:**
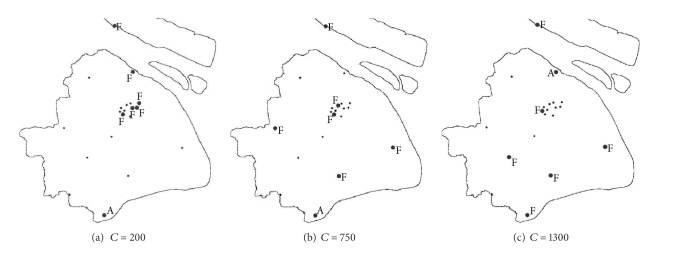
The facility location “F” and the district attacked “A” in equilibrium when *m* = 6 under different scenarios.

For fixed facility number, when the resources at each facility increase, the equilibrium solutions will vary, as Figure 3 shows. When the total resources are insufficient, once a district is attacked, more than one facility has to be involved. Then, the concentration mode of the resources could ensure the quick response to districts with more populations. The terrorist will attack Jinshan, which has less populations but is far away from all the terror response facilities, and the state will establish facilities at Pudong, Luwan, Xuhui, Yangpu, Baoshan, and Chongming. The state loss is mainly from the shipment delay of the resources. When the total resources are moderate, a single facility could satisfy the demand of some districts, and the facility distribution tends to be decentralized to improve the coverage. The terrorist will still attack Jinshan. When the total resources are abundant, a single facility could meet most demands, and the facility distribution will be more dispersed to improve the coverage further. In this case, the terrorist will attack Baoshan, which has more populations, and the state will establish facilities at Putuo, Jinshan, Songjiang, Nanhui, Fengxian, and Chongming. The state loss comes more from the affected populations.

**Figure 4:**
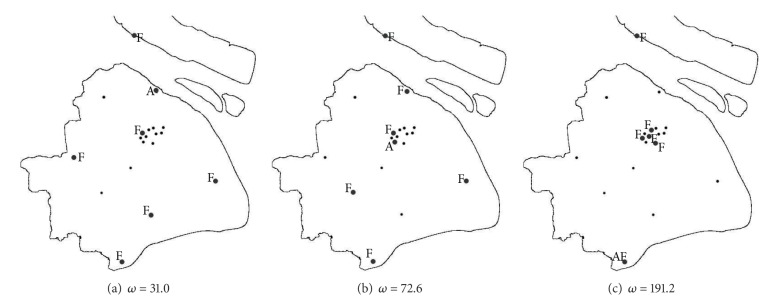
The facility location “F” and the district attacked “A” in equilibrium when *C* = 200, *m* = 6.

We consider a case that all districts have identical population, that is, *ω*_*i*_ = *ω*, for all  *i* ∈ *V*, to check the sensitivity of the equilibrium solution to the variations of the node weights. Let the weights be 31.0, 72.6, and 191.2, respectively, which correspond to the lowest, highest, and average of the populations of all districts. The state has a total of 200 resources, dispersing in 6 facilities. As we can see from Figure 4, the equilibrium solutions are not related to population quantity but with the population distribution, namely, the network structure. Also, the results are much different from the cases when the populations vary across the districts. When *ω* = 31.0, there are relatively many resources at each facility, and the distribution mode of the facilities tends to be decentralized; when *ω* = 72.6, 3 facilities have to be involved in the response, and the terrorist attacks the district which has more populations and faraway from all facilities; when *ω* = 191.2, all the 6 facilities must be involved. The problem for the state is equivalent to the *p*-median problem, and the terrorist will attack Jinshan.

**Figure 5:**
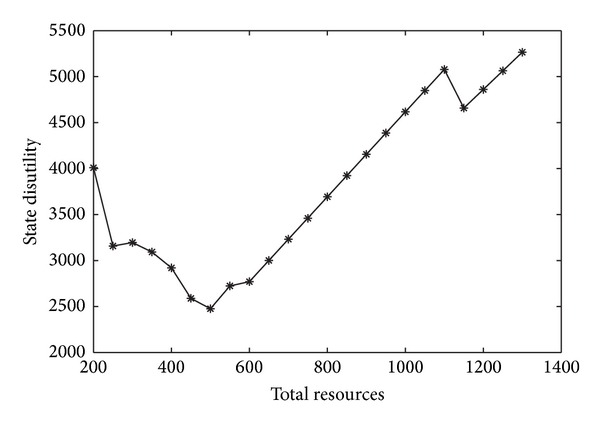
The relationship between total resources and state disutility when *m* = 6.

In practical, the state could determine the most cost-effective total resource according to the tradeoff curve between the total resources and the state disutility. For example, when *m* = 6, the tradeoff curve is shown in Figure 5. Obviously, the state should prepare 500 resources in the sense of cost efficiency.

## 6. Conclusions

In this paper, we introduced the capacity constraints into the game between the state and the terrorist. The government locates terror response facilities under capacity constraints first. The terrorist observes the distribution of the facilities then chooses the weakest target to attack. The government, who knows that its move in the game is the common knowledge, must place itself in its opponents' position before deciding the appropriate strategic response. An integer programming was proposed to solve the sequential game when the facility number is fixed. A case study was provided to analyze the strategic interaction of both sides. The equilibrium results showed that resource capacity did affect the action of the state and the terrorist. Centralized mode or dispersed mode for facility distribution depended on the total resources the state has.

The proposed approach can be of interest for modelling and solving more general response facility location problems under deliberate attack. The assumption that all the resources will be shipped as a whole may be limited to a certain extent. Besides, in real life, two or more bombings or suicide terrorist attacks usually occur simultaneously or sequentially, for example, the tragedy in Madrid. Moreover, the assumption that a strike on one city has no effect on other cities may be not always valid when biological or chemical attacks take place. This is beyond the scope of this paper and is the subject of ongoing research.

## Figures and Tables

**Table 1:** The state disutility under different scenarios.

*C* = 200	Facility number	1	2	3	4	5	6	7	8	9	10
	State disutility	12560	4770	5075.9	5045	4176	4009.3	3330	3702.2	3694.1	3472
	Facility number	11	12	13	14	15	16	17	18	19	
	State disutility	3336.1	3792.6	3497.3	3409.1	3742.6	3937.5	4060.4	4436.7	4899.3	

*C* = 750	Facility number	1	2	3	4	5	6	7	8	9	10
	State disutility	47100	17888	8629.3	6468.8	4455	3459	2402.4	2100	1864.8	1680
	Facility number	11	12	13	14	15	16	17	18	19	
	State disutility	2989.3	2743	2529.4	2348.8	2184.5	2053.1	1938.2	1823.2	1724.6	

*C* = 1300	Facility number	1	2	3	4	5	6	7	8	9	10
	State disutility	81640	31005	14957	10433	7202	5259.7	4164.2	3185	2583	1612
	Facility number	11	12	13	14	15	16	17	18	19	
	State disutility	1100.2	651.3	600.6	1161.9	1080.6	1454.4	1372.9	2489.2	2989.3	

**Table 2:** The 19 districts and their population sizes.

Node	District	Population size (×10^4^)
1	Pudong	191.2
2	Huangpu	60.6
3	Luwan	31.2
4	Xuhui	89.2
5	Changning	61.1
6	Jing'an	31.0
7	Putuo	86.3
8	Zhabei	69.5
9	Hongkou	79.0
10	Yangpu	107.7
11	Baoshan	83.1
12	Minhang	88.6
13	Jiading	53.8
14	Jinshan	52.1
15	Songjiang	54.3
16	Qingpu	45.7
17	Nanhui	73.4
18	Fengxian	51.6
19	Chongming	69.7

**Table 3:** The shortest distance matrix between the districts.

Districts	Pudong	Huangpu	Luwan	Xuhui	Changning	Jing'an	Putuo	Zhabei	Hongkou	Yangpu	Baoshan	Minhang	Jiading	Jinshan	Songjiang	Qingpu	Nanhui	Fengxian	Chongming
Pudong	0	6.3	7.6	12.4	12.4	10.3	16.4	9.8	6.5	6.9	24.3	22.7	36.0	66.9	41.6	43.1	35.8	40.6	67.3
Huangpu	6.3	0	2.3	7.0	6.1	3.9	10.1	3.4	4.7	5.7	21.2	17.3	29.7	65.1	36.3	37.1	42.2	39.7	63.0
Luwan	7.6	2.3	0	5.0	5.2	3.3	9.3	3.7	7.1	8.1	23.4	15.3	29.5	63.1	34.2	35.7	42.7	37.4	63.4
Xuhui	12.4	7.0	5.0	0	4.0	4.7	8.8	7.8	12.0	13.3	28.0	10.2	29.9	57.3	30.0	32.1	43.6	34.8	67.8
Changning	12.4	6.1	5.2	4.0	0	2.3	5.0	4.8	10.0	11.3	25.1	14.3	26.1	61.4	32.2	31.6	46.5	38.9	64.7
Jing'an	10.3	3.9	3.3	4.7	2.3	0	6.1	2.6	7.4	8.5	22.7	15.0	27.3	62.8	33.9	33.8	44.8	38.8	62.7
Putuo	16.4	10.1	9.3	8.8	5.0	6.1	0	7.1	12.3	14.2	24.3	17.7	21.3	63.5	34.1	33.2	50.8	41.7	62.8
Zhabei	9.8	3.4	3.7	7.8	4.8	2.6	7.1	0	5.2	7.0	20.6	18.0	26.0	65.8	37.0	36.6	45.4	40.9	60.6
Hongkou	6.5	4.7	7.1	12.0	10.0	7.4	12.3	5.2	0	2.4	17.9	22.3	29.8	70.1	41.3	41.5	42.4	44.5	60.8
Yangpu	6.9	5.7	8.1	13.3	11.3	8.5	14.2	7.0	2.4	0	18.9	23.6	31.9	70.8	42.5	42.8	40.3	44.5	61.9
Baoshan	24.3	21.2	23.4	28.0	25.1	22.7	24.3	20.6	17.9	18.9	0	38.3	22.9	86.2	57.2	53.3	54.8	60.8	43.8
Minhang	22.7	17.3	15.3	10.2	14.3	15.0	17.7	18.0	22.3	23.6	38.3	0	34.6	47.7	19.7	29.1	44.6	24.7	78.1
Jiading	36.0	29.7	29.5	29.9	26.1	27.3	21.3	26.0	29.8	31.9	22.9	34.6	0	78.0	43.1	32.9	71.6	58.1	54.3
Jinshan	66.9	65.1	63.1	57.3	61.4	62.8	63.5	65.8	70.1	70.8	86.2	47.7	78.0	0	38.8	57.0	60.5	27.7	125.7
Songjiang	41.6	36.3	34.2	30.0	32.2	33.9	34.1	37.0	41.3	42.5	57.2	19.7	43.1	38.8	0	19.6	59.4	34.7	96.0
Qingpu	43.1	37.1	35.7	32.1	31.6	33.8	33.2	36.6	41.5	42.8	53.3	29.1	32.9	57.0	19.6	0	73.1	52.6	86.3
Nanhui	35.8	42.2	42.7	43.6	46.5	44.8	50.8	45.4	42.4	40.3	54.8	44.6	71.6	60.5	59.4	73.1	0	34.5	99.2
Fengxian	40.6	39.7	37.4	34.8	38.9	38.8	41.7	40.9	44.5	44.5	60.8	24.7	58.1	27.7	34.7	52.6	34.5	0	100.7
Chongming	67.3	63.0	63.4	67.8	64.7	62.7	62.8	60.6	60.8	61.9	43.8	78.1	54.3	125.7	96.0	86.3	99.2	100.7	0
